# Natural history of MRI brain volumes in patients with neuronal ceroid lipofuscinosis 3: a sensitive imaging biomarker

**DOI:** 10.1007/s00234-022-02988-9

**Published:** 2022-06-14

**Authors:** Jan-Niklas Hochstein, A. Schulz, M. Nickel, S. Lezius, M. Grosser, J. Fiehler, J. Sedlacik, U. Löbel

**Affiliations:** 1 Department of Diagnostic and Interventional Neuroradiology, University Medical Center Hamburg-Eppendorf, Lokstedter Weg 104, 20251 Hamburg, Germany; 2 Department of Pediatrics, University Medical Center Hamburg-Eppendorf, Hamburg, Germany; 3 Department of Biometrics, University Medical Center Hamburg-Eppendorf, Hamburg, Germany; 4 Department of Radiology, Great Ormond Street Hospital for Children, London, UK

**Keywords:** Neuronal ceroid lipofuscinosis, CLN3 disease, Natural disease course, Brain volumetry, JNCL

## Abstract

**Purpose:**

Grey matter (GM) atrophy due to neuronal loss is a striking feature of patients with CLN3 disease. A precise and quantitative description of disease progression is needed in order to establish an evaluation tool for current and future experimental treatments. In order to develop a quantitative marker to measure brain volume outcome, we analysed the longitudinal volumetric development of GM, white matter (WM) and lateral ventricles and correlated those with the clinical course.

**Methods:**

One hundred twenty-two MRI scans of 35 patients (21 females; 14 males; age 15.3 ± 4.8 years) with genetically confirmed CLN3 disease were performed. A three-dimensional T1-weighted sequence was acquired with whole brain coverage. Volumetric segmentation of the brain was performed with the FreeSurfer image analysis suite. The clinical severity was assessed by the Hamburg jNCL score, a disease-specific scoring system.

**Results:**

The volumes of supratentorial cortical GM and supratentorial WM, cerebellar GM, basal ganglia/thalamus and hippocampus significantly (*r* =  − 0.86 to − 0.69, *p* < 0.0001) decreased with age, while the lateral ventricle volume increased (*r* = 0.68, *p* < 0.0001). Supratentorial WM volume correlated poorer with age (*r* =  − 0.56, *p* = 0.0001). Supratentorial cortical GM volume showed the steepest (4.6% (± 0.2%)) and most uniform decrease with strongest correlation with age (*r* =  − 0.86, *p* < 0.0001). In addition, a strong correlation with disease specific clinical scoring existed for the supratentorial cortical GM volume (*r* = 0.85, *p* =  < 0.0001).

**Conclusion:**

Supratentorial cortical GM volume is a sensitive parameter for assessment of disease progression even in early and late disease stages and represents a potential reliable outcome measure for evaluation of experimental therapies.

**Supplementary Information:**

The online version contains supplementary material available at 10.1007/s00234-022-02988-9.

## Introduction

Neuronal ceroid lipofuscinoses (NCLs) are the most common inherited degenerative brain disorders of childhood [1–3]. NCLs are incurable lysosomal storage disorders, and lysosomal accumulation of auto-fluorescent storage material, called ceroid lipofuscin, can be detected in almost all tissues [2]. The organs most dramatically affected are brain and retina. The brain undergoes a massive atrophy of neurons. Typical symptoms comprise progressive visual loss, psychomotor deterioration and epilepsy [3]. Based on their genetic defects, 13 different NCLs are classified (NCL1-14) [4–6].

One of the most prevalent NCL forms in Northern European countries is caused by mutations in the *CLN3* gene (CLN3 disease, also called juvenile NCL or Batten disease) [7–9]. CLN3 disease typically starts at around 4 to 6 years of age with progressive retinopathy leading to vision loss and blindness. After several years, dementia, epilepsy and loss of motor function ensue [10, 11]. The diagnostic hallmark of this NCL type is conspicuous vacuoles in the cytoplasm of lymphocytes.

*CLN3* encodes a polytopic membrane protein of 438 amino acids [3, 4, 7, 12]. To date, more than 40 different mutations in the *CLN3* gene have been described and are summarized in a mutation database [4–6, 13]. CLN3 disease is inherited autosomal-recessively, and 85% of CLN3 patients are homozygous for a 1-kb deletion that causes the loss of exons 7 and 8 [3, 12, 14]. Although the CLN3 protein is well conserved from yeast to humans, its function is still not fully understood. There are reports that CLN3 is involved in lysosomal acidification, arginine import, autophagy and apoptosis [3, 12, 14].

Even though pathomechanism and protein function in CLN3 are still needed to be further investigated, first, experimental treatments are being developed. Currently, an open-label phase I/II trial in patients with CLN3 disease (clinicaltrials.gov identifier NCT03770572) to assess intrathecal administration of AAV9-mediated gene therapy is ongoing. In addition, multiple pharmacological approaches such as gene editing, immunomodulation or improving lysosomal biogenesis by targeting transcription factor EB (TFEB) are tested in preclinical models, and clinical trials are in preparation [15]. The advent of new therapeutic strategies and their evaluation in clinical studies requires quantitative markers to describe the natural disease course [16]. Longitudinal natural history studies in CLN3 disease are challenging as the disease progression may extend over two decades with variability in phenotypes independent from the underlying genotype [15]. Disease-specific clinical rating scales such as the Hamburg JNCL score, which can be applied prospectively and retrospectively, are important to describe the long disease course. However, they are limited to significant loss of function (e.g., ambulation, language skills) [11]. Therefore, additional, more sensitive outcome measures are needed in order to assess treatment efficacy over a shorter period of time for potential future experimental therapies.

MRI allows quantitative assessment of the overall brain volume or individual brain structures using high-resolution T1-weighted imaging and post-processing. This has previously been used for CLN2 disease [17, 18]. Grey matter (GM) structures in particular show consistent loss of volume across the patient cohort with increasing age and can therefore be used as a sensitive biomarker for the natural course of the disease [17]. The decline of supratentorial cortical GM has previously been used as an outcome parameter for the first enzyme replacement therapy (cerliponase alfa, FDA-approved therapy) [19].

Brain atrophy has long been known to be a feature in CLN3 patients [20, 21]. However, studies on longitudinal brain volumetry in CLN3 patients are scarce. One study describes six genetically confirmed CLN3 patients at two time points [22], the other eight CLN3 patients at two time points, focusing mainly on hippocampal volume [23]. Correlation of the patient’s clinical disease course with the brain volumes is not available. Therefore, the purpose of this study was to describe the natural history of the disease course and develop a quantitative MRI volumetry-based biomarker for the evaluation of potential therapies for CLN3 patients. Since CLN3 disease causes neuronal loss [3, 12, 14], we expect GM volumes to be better suited as biomarker compared to white matter (WM) regions and CSF volumes which are only indirectly affected.

## Materials and methods

### Study design and participants

This is a prospective study approved in 2005. Quantitative volumetric MRI scans were acquired in addition to clinical routine consultation at our hospital. These data were collected for patients with genetically confirmed CLN3 disease.

The study protocol of this observational study was approved by the local ethical committee of the Ärztekammer Hamburg (PV7215), and informed consent was obtained for all patients prior to enrolment. The principles of the Declaration of Helsinki were followed.

Patients included in this cohort received at least one brain MRI scan between 2006 and 2016. Further on in the process, five patients were excluded (one family withdrew consent to use their data in studies, three patients did not have the sequences needed for volumetric analysis, and MRI quality was too low for one patient). From the patients enrolled, another 12 MRIs had to be excluded because of insufficient quality (*n* = 7) or missing imaging sequences (*n* = 5).

### Imaging

Imaging was performed on a 1.5 Tesla MRI (*n* = 116), but a 3 Tesla scanner was occasionally used when 1.5 T was unavailable (*n* = 6). One hundred eight scans were done at our hospital (96 on the same Siemens Avanto MRI, 12 on a Siemens Sonata MRI). The remaining 14 scans were performed on variable scanners in different hospitals so that families did not have to travel across the country for their imaging.

For volumetric analysis, a three-dimensional (3D) T1-weighted sequence was used. Imaging parameters varied slightly between the different scanners and changed on the Avanto scanner in 2012. A total of 55 MRIs were performed with the following imaging parameters: repetition time: 2280 ms, inversion time: 1000 ms, echo time: 3.64 ms, flip angle: 8°, acquisition matrix: 256 × 256, image voxel size: 0.5 × 0.5 × 1 mm^3^, whole-brain coverage on the sagittal plane. Before 2012, these parameters were different for 47 MRIs: repetition time: 1900 ms, inversion time: 1100 ms, echo time: 2.97 ms, flip angle: 15°, acquisition matrix: 256 × 176, image voxel size: 0.5 × 0.5 × 1 mm^3^, whole-brain coverage on axial plane. For additional information on follow-up times, imaging parameters and scanner variability, please see Table S1 of the supplement.

### Clinical scoring

Patients who had MR imaging performed at our hospital were also assessed clinically using clinical scoring for CLN3 disease [11]. Originally, the scale adds up to a total of 15 points in 5 categories: motor, vision, language, intellect and epilepsy. Each category ranks from 0 to 3 with 3 points representing a healthy, age-appropriate score and 0 points referring to no residual function. Since the epilepsy score is strongly influenced by each patient’s anticonvulsive medication, we excluded it from the total clinical score which therefore resulted in a maximum score of 12. All clinical scores used for correlation with MRI volumes were recorded in our NCL clinic by colleagues with significant experience in clinical assessment of NCL patients.

### Volumetric analysis

**Fig. 1:**
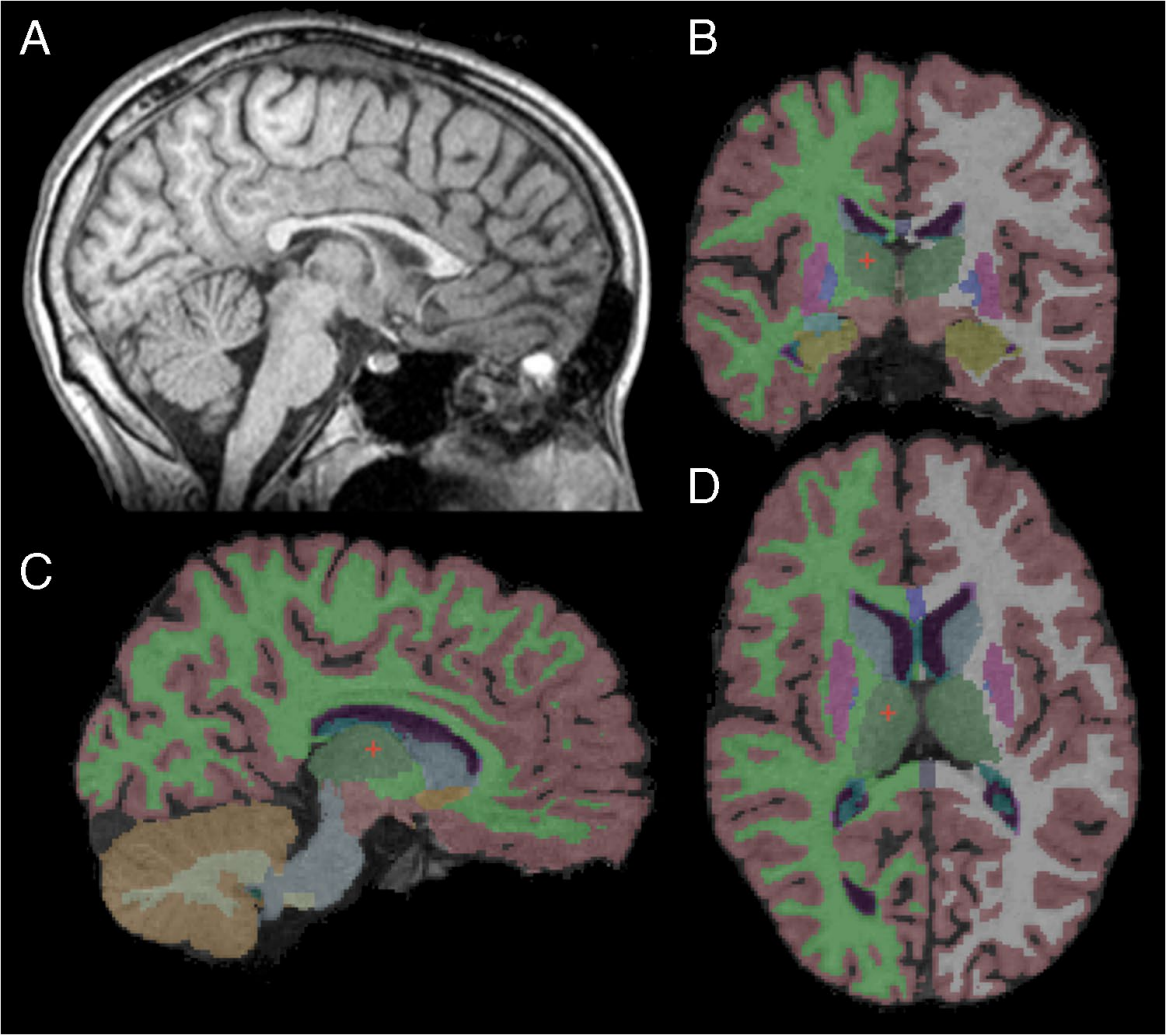
Segmentation of brain structures. **A** Midline sagittal image of the T1 MPRAGE sequence used for volumetric analysis. **B**–**D** Segmentation results using FreeSurfer in sagittal, coronal and axial planes

Brain segmentation was done using the FreeSurfer Image analysis suite Version 5.3.0 for each 3D T1 MRI data set [24, 25]. The FreeSurfer software extracts the brain tissue from surrounding extracranial tissue, performs a Talairach transformation, corrects deformations, determines the GM-WM interface and finally segments the brain into regions of interest (Fig. 1). All segments were visually reviewed. Prior to the final analysis, various different segmentation settings were used to determine the best parameters (e.g. performing the skull stripping with FSL brain extraction tool, using different segmentation settings or tools of the FreeSurfer program). Based on visual assessment, we found that the default FreeSurfer options gave the most consistent results compared to other options for segmentation. From all the segmented brain regions in the FreeSurfer output, we first decided to closer analyse the supratentorial cortical GM (consisting of supratentorial cortex, but excluding deep GM structures) since for CLN2 disease, it proved to be the best suitable biomarker [17]. We also included basal ganglia/thalamus (sum of putamen, pallidum, caudate nucleus and thalamus), cerebellar cortex and supratentorial WM volume; however, small or no changes have previously been reported for WM regions [17, 22]. For comparison with previous studies, hippocampal and ventricular volumes were segmented [17, 26].

### Statistical analysis

MRI volumes were correlated with patient age and clinical scores using Pearson’s correlation coefficient. In addition, linear models (lm-function) with age and clinical scores were fitted in R 3.6.2 (The R project for statistical computing) [24, 25] after calculating the logarithm of brain volumes. Patient’s sex, genetic mutation (1 = [c.462-677del,c.462-677del]; 2 = [c.1054C > T,c.462-677del]; 3 = [c.883G > 4,c.883G > 4]; 4 = [c.105G > A,c.222 + 5 > C and 5 = c.1054C > T, deletion (1,2 kb) Intron 13) and scan groups (1 = UKE pre 2012; 2 = UKE after 2012; 3 = all other scans) were used as covariates. Results were considered significant after Bonferroni correction with *p* ≤ 0.008 to account for multiple testing of the six brain regions. Patient no. 03 was excluded from all statistical analyses due to an atypical phenotype and is discussed separately.

## Results

### Patients

Our final cohort included 35 genetically confirmed CLN3 patients (21 females, 14 males; mean age: 15.3 years; youngest patient: 7 years, oldest patient: 29 years). Twenty-seven patients were homozygous for the most common *CLN3* mutation (c.462-677del/c.462-677del), 3 patients were compound heterozygous (c.1054C > T / c.462-677del), one patient was homozygous for c.883G > 4 and two patients compound heterozygous for different mutations (c.105G > A / c.222 + 5G > C and c.1054C > T/deletion (1,2 kb) Intron 13). In two patients, diagnosis was based on detection of characteristic lysosomal storage material by electron microscopy in a skin biopsy and classic clinical presentation. Genetic analysis had only been performed for detection of the frequent 1.2 kb deletion which could not be confirmed. Please see Supplement Table S1 for more details on patient demographics and genetics.

### Clinical scoring

The total clinical score at the time of each MRI examination is displayed in Supplement Table S2. None of the patient had visual scores of 3; therefore, the highest total score achieved was 11. Overall, the score showed a constant decline over the study period of 8 years, but with great variability: for example, one 11-year-old patient (#17) has a total score of 5, while another 11-year-old (#4) has a clinical score of 10). In addition, some patients maintained a stable score over a long period of time (e.g., no. 34 from age 17 to 21, score 6; no. 33 from age 21 to 24, score 3; no. 01 from age 12 to 15, score 6; no. 22 from age 15 to 18, score 4). Both, patient no. 11 and patient no. 26, reach a score of 0. Of note, patient no. 11 had four follow-up scans demonstrating a constant decline in supratentorial cortical GM volume at corresponding clinical scores of 0.

### Correlation of MRI volumetry with patient age and covariates

The lm model showed no significant effect of covariates (i.e., genetics, scan group) for the investigated brain regions (e.g., for the correlation with supratentorial cortical GM with genetic mutation, *p* = 0.205; for the correlation with scan group, *p* = 0.946; see Supplement Table S3). Therefore, the linear model was fitted between the investigated brain region volumes and patient age with patient sex as the only covariate.

**Fig. 2:**
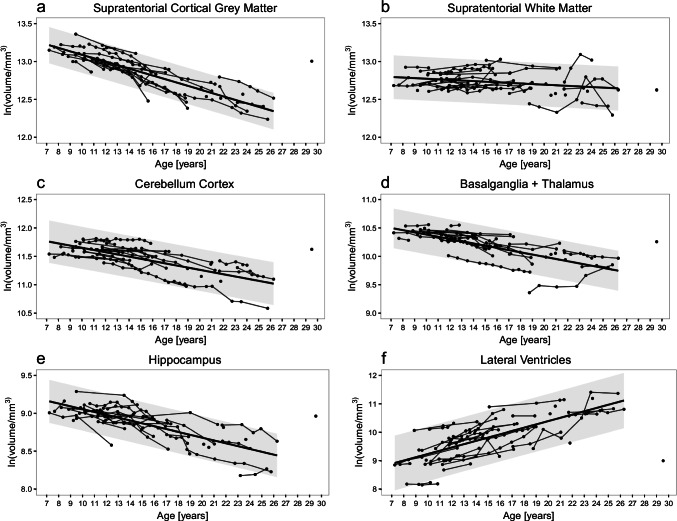
Age-related development of brain volumes. A decrease in grey and white matter volumes with age is noted (**A–E**) which is most prominent in supratentorial cortical grey matter (**A**). The size of the lateral ventricles significantly decreases with age (**F**)

A strong decline in brain volume with age was identified for all regions (supratentorial cortical GM, cerebellar GM, hippocampus, basal ganglia/thalamus, supratentorial WM), while the size of the lateral ventricles increased (Table 1; Fig. 2). The most uniform decline visually — but also statistically — was observed for the supratentorial cortical GM volumes with sex as cofactor (*r* = 0.86, *p* < 0.001). Weaker, but still statistically significant correlations were seen for supratentorial WM volumes (*r* = 0.56, *p* < 0.001).

**Table 1:** Results. Percentage of volume loss per year for all brain regions and results of correlation with patient age and clinical scores (with covariate sex)

Region of interest	Volume change (per year)			Correlation (*r*-values)	
	%	Standard deviation		With age	With Hamburg jNCL Total score
		Upper limit	Lower limit		
Supratentorial cortical GM	− 4.60	− 4.40	− 4.78	0.862*	0.850*
Female supratentorial cortical GM Male supratentorial cortical GM	− 4.68 − 4.47	− 4.46 − 4.12	− 4.90 − 4.83	0.932* 0.768*	0.887* 0.801*
Supratentorial WM	− 0.97	− 0.74	− 1.21	0.561*	0.563*
Cerebellar cortex	− 3.95	− 3.61	− 4.28	0.692*	0.750*
Basal ganglia/thalamus	− 3.87	− 3.55	− 4.20	0.691*	0.805*
Hippocampus	− 3.83	− 3.60	− 4.07	0.802*	0.787*
Lateral Ventricles	+ 12.06	+ 13.13	+ 11.02	0.683*	0.606*

^*^All *p*-values < 0.00001, except supratentorial WM: *p*-values: with age = 0.0001, with clinical score = 0.00012

For supratentorial cortical GM volume, the annual loss was 4.6% per year (95%-prediction interval: 4.4–4.8%). This was similar, but slightly less prominent for all other GM regions (hippocampus: 3.8%, cerebellar cortex: 4.0%, basal ganglia/thalamus: 3.9%). Supratentorial WM volumes remained relatively stable over the observed time interval with an annual decline of 1.0% (95%-prediction interval: 0.74%, 1.2%). The lateral ventricles showed an annual increase in size of 12% (prediction interval: 11.0–13.1%). The effect of sex was between 15.1% for supratentorial WM and 3.51% for the lateral ventricles.

**Fig. 3:**
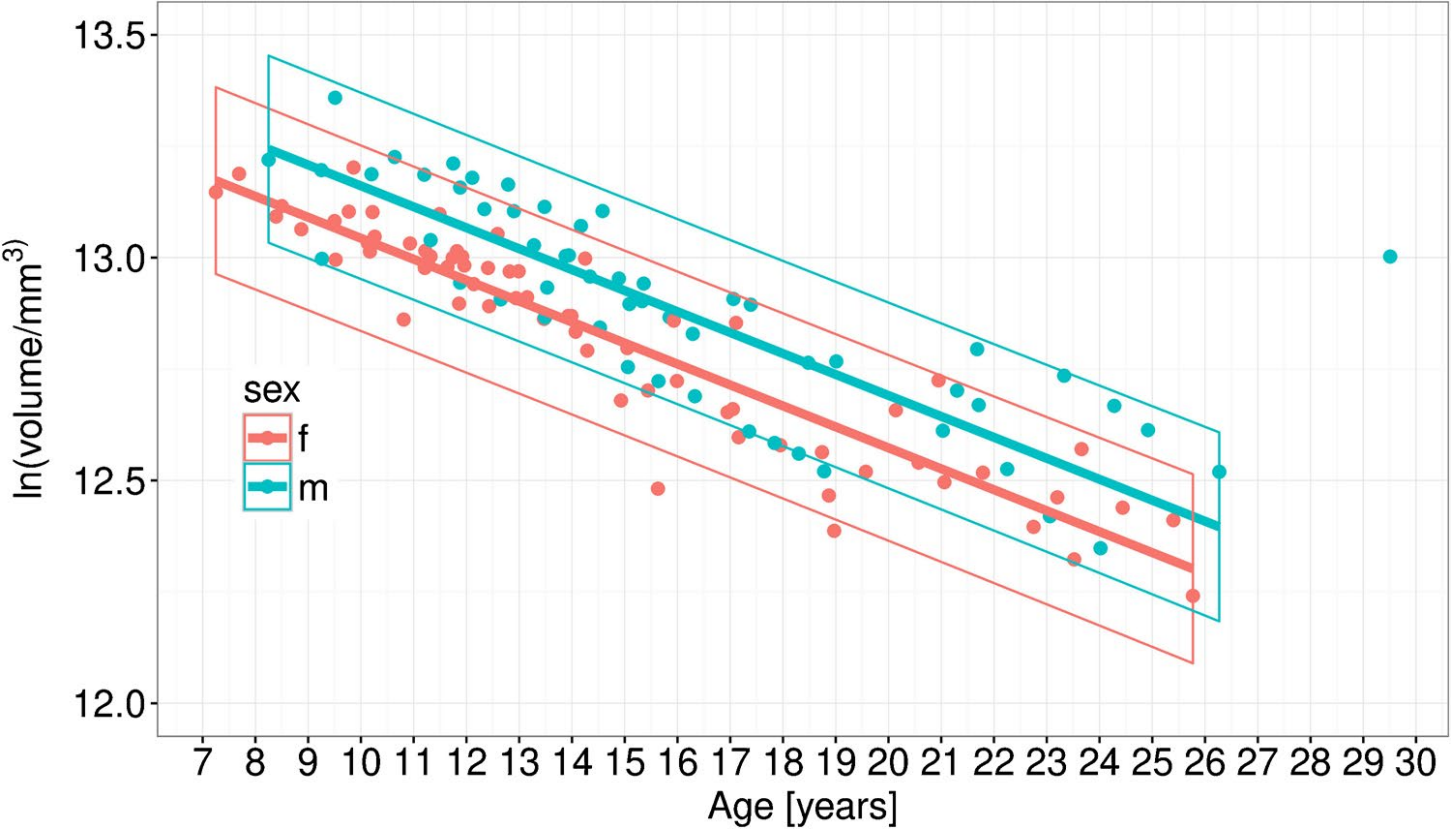
Sex-related differences in brain volumes. Female patients are depicted in red, male patients in blue colour

The correlation of patient age with GM volumes is highly significant for both, girls and boys (female *r* = 0.93, male *r* = 0.77) and is displayed in Fig. 3. The annual decline in volume was 4.7% (prediction interval: 4.6–4.8%) and 4.5% (prediction interval: 4.3–4.7%) for girls and boys, respectively.

### Correlation MRI volumetry with clinical scoring

**Fig. 4:**
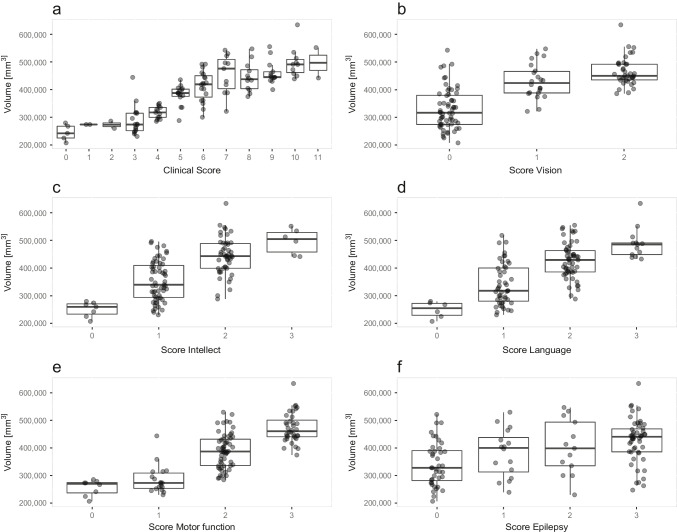
Relationship of supratentorial cortical grey matter volume with clinical scoring. The supratentorial cortical GM volume (*y*-axis) is plotted against **A** the Hamburg JNCL total score as well as the individual scores for **B** vision, **C** intellect, **D** language, **E** motor function and **F** epilepsy

The total clinical score at the time of each MRI examination in correlation to supratentorial cortical GM volumes is depicted in Fig. 4. The correlation is highly significant (*r* = 0.85, *p* < 0.0001). The correlation for other GM regions and the lateral ventricles similar, but slightly less significant (Table 1, last column).

The correlation of brain volumes with the total clinical score shows a marginally stronger correlation compared to the correlation of brain volumes with patient age in some regions while in others, the correlation with age is slightly stronger (e.g., for supratentorial cortical GM, correlation with age is slightly better; for cerebellum cortex, correlation with clinical scores is marginally stronger).

Similar to the total clinical score, the single scores for motor, visual, language, intellect and epilepsy were plotted against the supratentorial cortical GM volume (Fig. 4). Generally, lower single clinical scores were observed in patients with lower brain volumes.

Of all single scores, the epilepsy score showed the highest variability with respect to supratentorial cortical GM, although higher epilepsy scores were still associated with higher brain volumes.

## Discussion

In this prospective MRI study of 35 genetically confirmed CLN3 patients, we found that volumes of all examined GM and WM structures significantly decreased with age. There was also a strong correlation with the clinical disease course described by the Hamburg JNCL score. Of all the regions studied, supratentorial cortical GM volumes showed the strongest, most uniform decline (4.6% per year) and strongest correlation with clinical score and patient age. Specific reasons for this finding have not been clearly identified, but is likely multifactorial and related to selective vulnerability [27]. The slower decline in supratentorial WM volumes is believed to be related to secondary white matter involvement, a finding that matches histopathological data and has been reported previously [17, 22].

Generally, clinical progression in CLN3 disease is slower compared to other NCL forms, spanning over more than two decades. Clinical scores may therefore remain unchanged over a significant period of time despite progressive atrophy on MRI [11, 28]. Specifically, clinical scoring may be limited in early and late stages of the disease. In early stages, vision loss is the predominant symptom, but scoring also includes other categories such as cognition, motor and language which may not yet be affected. In late stages, once a score of zero has been reached, further disease progression cannot be assessed. In contrast, MRI showed continuous decrease in brain volume across the entire observation period despite stable clinical scores (e.g. patient no. 04 and no. 13 showed decreasing brain volumes with stable clinical scores of 10; patient no. 11 showed a constant decline in volume with corresponding clinical scores of 0). Therefore, automated brain volumetry provided an objective assessment of disease progression in NCL patients. In contrast to clinical scoring, which should ideally be done in the same centre by experienced clinicians, we were able to include a small number of MRI datasets from different centres using similar imaging parameters and the same segmentation software. Statistical analysis showed no effect of scanner types on volumetric results.

Interestingly, patient no. 03 showed a very distinct clinical disease course albeit having the most commonly seen genetic mutation (homozygous 1-kb deletion). Based on the atypical clinical phenotype, the diagnosis could only be made at the age of 27 years. Reasons for this atypical phenotype have been studied by Lebrun et al. where this patient represents patient no. 04 in the assessments, but potential modifier genes are still under investigation [15].

We evaluated whether the different genetic mutations have an impact on the brain volumes. Patients with five different genetic mutations in the *CLN3* gene leading to CLN3 disease were included in this study. The different mutations do not seem to have an impact on the rate of volume decline. However, the number of patients in individual groups is too small to obtain significant results.

We also analysed sex as a covariate influencing the uniformity of the volume loss. Similar to findings for healthy children [29], we saw a 12.6% smaller supratentorial cortical GM volume in girls compared to boys. However, brain volumes declined at a similar rate, decreasing at an annual rate of 4.7% and 4.5% for girls and for boys, respectively (Fig. 3). In contrast, Cialone et al. suggest that girls might experience a more severe disease course [30].

Even in the healthy population, brain volumes change during childhood and adolescence [31–33]. GM structures show a small relative decrease in volume while WM volumes increase during adolescence [31–33]. Brain volume loss in CLN3 patients was much more rapid compared to the healthy population.

Previous studies in smaller CLN3 cohorts reported an annual volume loss of 3.3% (SD 1.9) and 2.4% for bilateral hippocampal volume and overall GM, respectively [22, 23]. In our study, the volume loss exceeds these findings, but for the hippocampal volume, it is within the standard deviation. Discrepant findings are likely due to longer study periods in our cohort, different regions of interest (global GM vs supratentorial cortical GM) and different segmentation software used [5].

Some limitations of the study need to be addressed. All GM regions of CLN3 patients studied declined in volume. However, artefacts caused by the segmentation software can influence the volumetric results. These issues are better counterbalanced in a large GM region than in small brain regions leading to a more uniform decline and a stronger correlation. Additionally, segmentation of deep GM and cerebellum is more challenging than the supratentorial cortical GM regions [34]. Therefore, we believe that the supratentorial cortical GM volume is better suited as biomarker compared to small GM regions such as hippocampus or basal ganglia/thalamus.

Although our patient cohort is relatively large, further studies are needed to assess very early disease stages (i.e. before development of clinical symptoms) and very late stages where our data was limited. Sequence parameters used for our MRI scans differed little from those recommended for FreeSurfer; however, differences in flip angles (suggested angle of 7° vs. 8–15° used) and repetition times (suggested 2700 ms vs. 1900–2280 ms used) were noted. Although our results showed strong correlations with patient age and clinical scoring, our data may have improved even further using the recommended imaging parameters. In addition, imaging parameters and scanners varied over time, and the consistent use of a single study protocol and MR scanner might further improve the data. However, we believe that the amount of volume loss studied is much larger than effects from using different scanners or slight variations in sequence parameters as statistical analysis did not show an influence of these factors on our results.

## Conclusion

In conclusion, automated longitudinal MRI volumetry is an objective and sensitive tool to observe progression in CLN3 disease. Progressive brain atrophy was seen in all analysed GM structures with a strong correlation to clinical scoring. Supratentorial cortical GM showed the most uniform decline and the strongest correlation with patient age and clinical disease course and would be the most useful region to objectively monitor natural disease progression. The data provided is important for the assessment of future therapies in patients with CLN3, especially since MRI volumetry is able to describe disease progression at early and late stages where clinical disease monitoring by scoring systems alone may not be sensitive enough.

## Supplementary Information

ESM 1 Figure S1: Genetics (PNG 294 kb)

ESM 1 Figure S2: Scan groups (PNG 147 kb)

High resolution image (EPS 72 kb)

ESM 3 (DOCX 20 kb)

ESM 4 (DOCX 28 kb)

ESM 5 (DOCX 16 kb)

## References

[1] Zeman W, Dyken P (1969). Neuronal ceroid-lipofuscinosis (Batten’s disease): relationship to amaurotic family idiocy?. Pediatrics.

[2] Haltia M (2003). The neuronal ceroid-lipofuscinoses. J Neuropathol Exp Neurol.

[3] Mole SE, Williams RE, Goebel H (2005). Correlations between genotype, ultrastructural morphology and clinical phenotype in the neuronal ceroid lipofuscinoses. Neurogenetics.

[4] Warrier V, Vieira M, Mole SE (2013). Genetic basis and phenotypic correlations of the neuronal ceroid lipofusinoses. Biochim Biophys Acta.

[5] Williams RE, Mole SE (2012). New nomenclature and classification scheme for the neuronal ceroid lipofuscinoses. Neurology.

[6] Kousi M, Lehesjoki AE, Mole SE (2012). Update of the mutation spectrum and clinical correlations of over 360 mutations in eight genes that underlie the neuronal ceroid lipofuscinoses. Hum Mutat.

[7] Claussen M, Heim P, Knispel J, Goebel HH, Kohlschütter A (1992). Incidence of neuronal ceroid-lipofuscinoses in West Germany: variation of a method for studying autosomal recessive disorders. Am J Med Genet.

[8] Uvebrant P, Hagberg B (1997). Neuronal ceroid lipofuscinoses in Scandinavia. Epidemiology and clinical pictures. Neuropediatrics.

[9] Moore SJ, Buckley DJ, MacMillan A (2008). The clinical and genetic epidemiology of neuronal ceroid lipofuscinosis in Newfoundland. Clin Genet.

[10] Schulz A, Kohlschütter A, Mink J, Simonati A, Williams R (2013). NCL diseases - clinical perspectives. Biochim Biophys Acta.

[11] Kohlschütter A, Laabs R, Albani M (1988). Juvenile neuronal ceroid lipofuscinosis (JNCL): quantitative description of its clinical variability. Acta Paediatr Scand.

[12] Rakheja D, Narayan SB, Bennett MJ (2008). The function of CLN3P, the batten disease protein. Mol Genet Metab.

[13] NCL Resource - a gateway for Batten disease. https://www.ucl.ac.uk/ncl-disease/mutation-and-patient-database/mutation-and-patient-datasheets-human-ncl-genes/cln3. Accessed 23 Oct 2021

[14] Cotman SL, Lefrancois S (2021). CLN3, at the crossroads of endocytic trafficking. Neurosci Lett.

[15] Mole SE, Anderson G, Band HA (2019). Clinical challenges and future therapeutic approaches for neuronal ceroid lipofuscinosis. Lancet Neurol.

[16] Kohlschütter A, Schulz A, Bartsch U, Storch S (2019). Current and emerging treatment strategies for neuronal ceroid lipofuscinoses. CNS Drugs.

[17] Löbel U, Sedlacik J, Nickel M (2016). Volumetric description of brain atrophy in neuronal ceroid lipofuscinosis 2: supratentorial gray matter shows uniform disease progression. Am J Neuroradiol.

[18] Dyke JP, Sondhi D, Voss HU (2016). Brain region-specific degeneration with disease progression in late infantile neuronal ceroid lipofuscinosis (CLN2 disease). AJNR Am J Neuroradiol.

[19] Schulz A, Ajayi T, Specchio N (2018). Study of intraventricular cerliponase alfa for CLN2 disease. N Engl J Med.

[20] Autti T, Raininko R, Vanhanen SL, Santavuori P (1996). MRI of neuronal ceroid lipofuscinosis I. Cranial MRI of 30 patients with juvenile neuronal ceroid lipofuscinosis. Neuroradiology.

[21] Järvelä I, Autti T, Santavuori P, Raininko R, Åberg L, Peltonen L (1997). Clinical and MRI findings in Batten disease – analysis of the major mutation. Ann Neurol.

[22] Autti TH, Hämäläinen J, Mannerkoski M (2008). JNCL patients show marked brain volume alterations on longitudinal MRI in adolescence. J Neurol.

[23] Tokola AM, Eero KS, Åberg LE, Autti T (2014). hippocampal volumes in juvenile neuronal ceroid lipofuscinosis: a longitudinal magnetic resonance imaging study. Pediatr Neurol.

[24] Reuter M, Schmansky NJ, Rosas HD, Fischl B (2012). Within-subject template estimation for unbiased longitudinal image analysis. Neuroimage.

[25] Han X, Jovicich J, Salat D (2006). Reliability of MRI-derived measurements of human cerebral cortical thickness: the effects of field strength, scanner upgrade and manufacturer. Neuroimage.

[26] Dyke JP, Sondhi D, Voss HU (2013). Assessment of disease severity in late infantile neuronal ceroid lipofuscinosis using multiparametric MR imaging. AJNR Am J Neuroradiol.

[27] Biswas A, Krishnan P, Amirabadi A (2020). Expanding the neuroimaging phenotype of neuronal ceroid lipofuscinoses. AJNR Am J Neuroradiol.

[28] Marshall FJ, de Blieck EA, Mink JW (2005). A clinical rating scale for Batten disease: reliable and relevant for clinical trials. Neurology.

[29] Ruigrok ANV, Salimi-Khorshidi G, Lai MC (2014). A meta-analysis of sex differences in human brain structure. Neurosci Biobehav Rev.

[30] Cialone J, Adams H, Augustine EF (2012). Females experience a more severe disease course in Batten disease. J Inherit Metab Dis.

[31] Wilke M, Krägeloh-Mann I, Holland SK (2007). Global and local development of gray and white matter volume in normal children and adolescents. Exp Brain Res.

[32] Giedd JN, Snell JW, Lange N (1996). Quantitative magnetic resonance imaging of human brain development: ages 4–18. Cereb Cortex.

[33] Giedd JN, Blumenthal J, Jeffries NO (1999). Brain development during childhood and adolescence: a longitudinal MRI study. Nat Neurosci.

[34] Fischl B, Salat D, Busa E (2002). Whole brain segmentation: automated labeling of neuroanatomical structures in the human brain. Neuron.

