# Supplementary figures and images for: Natural history of MRI brain volumes in patients with neuronal ceroid lipofuscinosis 3: a sensitive imaging biomarker
Source: Neuroradiology. 2022 Jun 14;64(10):2059–67. doi: 10.1007/s00234-022-02988-9 (PMC9474504; doi:10.1007/s00234-022-02988-9)

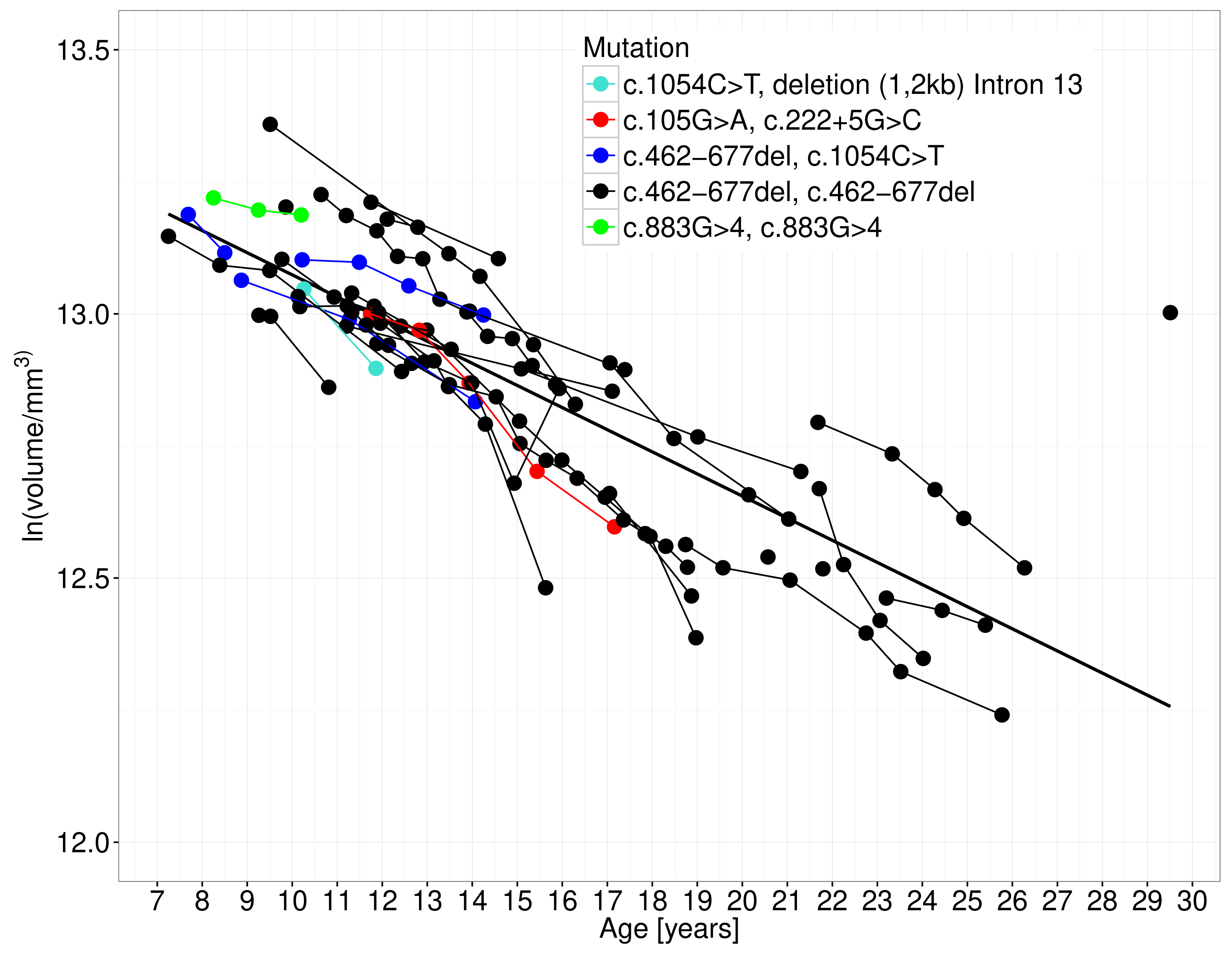

Supplement: Supplementary file 1 — Figure S1: Genetics (PNG 294 kb) [file 234_2022_2988_Fig5_ESM.png]
