# Supplementary material for: Natural history of MRI brain volumes in patients with neuronal ceroid lipofuscinosis 3: a sensitive imaging biomarker
Source: Neuroradiology. 2022 Jun 14;64(10):2059–67. doi: 10.1007/s00234-022-02988-9 (PMC9474504; doi:10.1007/s00234-022-02988-9)
Supplement: Supplementary file 4 — (DOCX 20 kb) [file 234_2022_2988_MOESM3_ESM.docx]

|  | N | | 2 SD |
| --- | --- | --- | --- |
| **Gender**  Male  Female  **Genetics**  c.462-677del, c.462-677del  c.462-677del, c.1054C>T  c.105G>A, c.222+5G>C  c.1054C>T, other intragenic deletion (1,2kb) Intron 13  c.883G>4, c.883G>4  no 1kb, no 1kb  **Number of MRIs per Patient**  1  2-5  6-10  >10  Total  **Average longitudinal time span per patient**  (=Time between first and last MRI of one Patient)  Patients with 2-5 MRIs  Patients with 6-10 MRIs  Patients with >10 MRIs  All Patients with 2 or more MRI  **Age**  Average Age  Youngest Patient at first MRI  Oldest Patient at last MRI  Longest longitudinal span (first to last MRI)  **Imaging Parameters**  UKE before 2012  UKE after 2012  Others  Patients with only MRIs UKE before 2012  Patients with MRIs only UKE after 2012  Patients with MRIs both UKE after and before 2012 and other Imaging Parameters  **MR-Scanner**  Patients with all MRI on Sonata Siemens 1,5T  Patients with all MRI on Avanto Siemens 1,5T  Patients with both/other scanners | 14  21  27  3  1  1  1  2  8  22  3  2  35  3,36 years  7 years  6,1 years  3,97 years  15.3 years  7.3 years  29.5 years  7,7 years  47 MRIs  55 MRIs  14MRIs  7 Patients  4 Patients  25 Patients  18 Patients  5 Patients  12 Patients | ± 3,69 years  ± 1,51 years  ± 2,26 years  ± 4,27 years  ± 4.8 years | |
