# Supplementary material for: Natural history of MRI brain volumes in patients with neuronal ceroid lipofuscinosis 3: a sensitive imaging biomarker
Source: Neuroradiology. 2022 Jun 14;64(10):2059–67. doi: 10.1007/s00234-022-02988-9 (PMC9474504; doi:10.1007/s00234-022-02988-9)
Supplement: Supplementary file 5 — (DOCX 28 kb) [file 234_2022_2988_MOESM4_ESM.docx]

| *Patient-ID* | *Age [years]* | *Supratentorial Cortical Grey Matter volume [mm³]* | *Hamburg jNCL Total Score* |
| --- | --- | --- | --- |
| Pat01 | 10,6 | 554546 | 9 |
| Pat01 | 11,2 | 532967 | 9 |
| Pat01 | 11,9 | 517778 | 8 |
| Pat01 | 12,3 | 493341 | 7 |
| Pat01 | 12,9 | 491069 | 6 |
| Pat01 | 13,3 | 454906 | 6 |
| Pat01 | 13,9 | 444070 | 6 |
| Pat01 | 14,3 | 424037 | 6 |
| Pat01 | 14,9 | 422181 | 6 |
| Pat01 | 15,3 | 401229 | 6 |
| Pat01 | 15,9 | 387076 | 5 |
| Pat04 | 8,9 | 471472 | 10 |
| Pat04 | 11,3 | 437822 | 10 |
| Pat04 | 14,1 | 374670 | 8 |
| Pat05 | 12,1 | 529279 | 7 |
| Pat05 | 12,8 | 521273 | 7 |
| Pat07 | 11,3 | 443695 | 9 |
| Pat07 | 11,8 | 448885 | 8 |
| Pat07 | 12,4 | 432300 | 8 |
| Pat07 | 13,0 | 429038 | 7 |
| Pat07 | 15,1 | 361150 | 6 |
| Pat07 | 16,0 | 335435 | 5 |
| Pat07 | 17,0 | 312729 | 4 |
| Pat07 | 18,0 | 290405 | 4 |
| Pat07 | 19,0 | 239661 | 3 |
| Pat09 | 15,1 | 398697 | 5 |
| Pat09 | 19,0 | 350512 | 4 |
| Pat09 | 21,3 | 328311 | 4 |
| Pat10 | 11,7 | 442337 | 9 |
| Pat10 | 12,8 | 428917 | 9 |
| Pat10 | 13,9 | 388156 | 7 |
| Pat10 | 15,4 | 328393 | 6 |
| Pat10 | 17,2 | 295659 | 3 |
| Pat11 | 18,7 | 285892 | 2 |
| Pat11 | 19,6 | 273619 | 1 |
| Pat11 | 21,1 | 267286 | 0 |
| Pat11 | 22,8 | 241796 | 0 |
| Pat11 | 23,5 | 224776 | 0 |
| Pat11 | 25,8 | 207096 | 0 |
| Pat12 | 9,5 | 633393 | 10 |
| Pat12 | 13,5 | 495816 | 7 |
| Pat12 | 14,2 | 475000 | 7 |
| Pat12 | 15,4 | 417404 | 6 |
| Pat12 | 16,3 | 372868 | 5 |
| Pat13 | 7,7 | 534033 | 10 |
| Pat13 | 8,5 | 496662 | 10 |
| Pat14 | 21,7 | 360301 | 6 |
| Pat14 | 23,3 | 339450 | 4 |
| Pat14 | 24,3 | 317242 | 4 |
| Pat14 | 24,9 | 300448 | 4 |
| Pat14 | 26,3 | 273578 | 1 |
| Pat15 | 10,3 | 463742 | 9 |
| Pat15 | 11,9 | 399052 | 9 |
| Pat16 | 7,3 | 512420 | 10 |
| Pat16 | 8,4 | 485119 | 8 |
| Pat16 | 9,5 | 480294 | 6 |
| Pat16 | 12,4 | 396705 | 5 |
| Pat17 | 12,0 | 434707 | 5 |
| Pat17 | 13,2 | 404760 | 5 |
| Pat17 | 13,5 | 385579 | 5 |
| Pat17 | 14,3 | 359107 | 3 |
| Pat17 | 15,6 | 263430 | 3 |
| Pat18 | 10,1 | 457154 | 10 |
| Pat18 | 10,2 | 448519 | 10 |
| Pat18 | 11,2 | 449236 | 9 |
| Pat18 | 11,6 | 433235 | 8 |
| Pat18 | 12,1 | 416887 | 7 |
| Pat20 | 17,1 | 314917 | 3 |
| Pat20 | 18,9 | 259410 | 2 |
| Pat22 | 11,9 | 418280 | 6 |
| Pat22 | 12,7 | 402860 | 5 |
| Pat22 | 13,5 | 386966 | 5 |
| Pat22 | 14,5 | 378161 | 5 |
| Pat22 | 15,1 | 346121 | 4 |
| Pat22 | 15,6 | 335342 | 4 |
| Pat22 | 16,3 | 324153 | 4 |
| Pat22 | 17,4 | 299538 | 4 |
| Pat22 | 17,8 | 291871 | 4 |
| Pat22 | 18,3 | 284972 | 4 |
| Pat22 | 18,8 | 273888 | 3 |
| Pat23 | 8,3 | 551044 | 11 |
| Pat23 | 9,3 | 538425 |  |
| Pat23 | 10,2 | 533515 |  |
| Pat24 | 11,3 | 460200 | 6 |
| Pat24 | 13,5 | 413723 | 5 |
| Pat25 | 21,8 | 273067 | 3 |
| Pat26 | 20,6 | 279255 | 0 |
| Pat27 | 13,9 | 444711 | 9 |
| Pat27 | 17,4 | 398072 | 8 |
| Pat29 | 10,2 | 490071 | 10 |
| Pat29 | 11,5 | 487847 | 9 |
| Pat29 | 12,6 | 466553 | 8 |
| Pat29 | 14,3 | 441480 | 6 |
| Pat30 | 11,2 | 432350 | 9 |
| Pat30 | 17,1 | 382155 | 5 |
| Pat31 | 21,0 | 335888 | 5 |
| Pat32 | 23,7 | 287875 | 5 |
| Pat33 | 21,7 | 317805 | 3 |
| Pat33 | 22,3 | 275271 | 3 |
| Pat33 | 23,1 | 247651 | 3 |
| Pat33 | 24,0 | 230479 | 3 |
| Pat34 | 17,1 | 403240 | 6 |
| Pat34 | 18,5 | 349457 | 6 |
| Pat34 | 21,0 | 300032 | 6 |
| Pat37 | 20,1 | 314159 | 3 |
| Pat38 | 9,8 | 490570 | 10 |
| Pat38 | 10,9 | 456730 | 9 |
| Pat38 | 11,9 | 443453 | 9 |
| Pat38 | 12,9 | 404021 | 8 |
| Pat38 | 14,0 | 388150 | 7 |
| Pat38 | 14,9 | 321113 | 7 |
| Pat38 | 15,9 | 384128 | 6 |
| Pat39 | 11,8 | 546522 | 8 |
| Pat39 | 14,6 | 491207 | 6 |
| Pat40 | 23,2 | 258352 | 3 |
| Pat40 | 24,4 | 252413 | 3 |
| Pat40 | 25,4 | 245439 | 3 |
| Pat42 | 9,3 | 441258 | 11 |
| Pat43 | 9,5 | 440379 | 8 |
| Pat43 | 10,8 | 385052 | 8 |
| Pat46 | 9,9 | 541607 | 7 |
| Pat03 | 29,5 | 443485 | 3 |
