# Supplementary material for: Natural history of MRI brain volumes in patients with neuronal ceroid lipofuscinosis 3: a sensitive imaging biomarker
Source: Neuroradiology. 2022 Jun 14;64(10):2059–67. doi: 10.1007/s00234-022-02988-9 (PMC9474504; doi:10.1007/s00234-022-02988-9)
Supplement: Supplementary file 6 — (DOCX 16 kb) [file 234_2022_2988_MOESM5_ESM.docx]

**Supplement Table S3:** Korrelation of the ROIs with patient age and Hamburg jNCL total score with and without covariates (sex, scangroups and genetics) and impact of the covariate sex on the volume of the ROIs in percent

|  | covariate sex | | covariate scangroups | | covariate genetics | | impact covariate sex |
| --- | --- | --- | --- | --- | --- | --- | --- |
|  | with age | with score | with age | with score | with age | with score |  |
|  | p | p | p | p | p | p |  |
| Supratentorial cortical GM | 1.577316e-07 | 3.957364e-04 | 9.460569e-01 | 4.077946e-01 | 2.050084e-01 | 2.879931e-02 | 12.62% |
| supratentorial WM | 4.881292e-10 | 4.468672e-09 | 3.072716e-01 | 2.322379e-01 | 1.399559e-01 | 3.097442e-01 | 15.1% |
| Cerebellum Cortex | 4.863668e-04 | 3.160977e-03 | 6.602366e-02 | 1.961885e-01 | 4.857383e-01 | 6.828386e-02 | 12.29% |
| Basalganglia | 2.237531e-01 | 8.135405e-01 | 8.091686e-02 | 1.022369e-01 | 8.997638e-01 | 4.339021e-01 | 4.07% |
| Hippocampus | 2.801512e-08 | 1.263138e-05 | 1.842702e-01 | 6.080138e-01 | 1.129820e-01 | 9.901480e-03 | 14.55% |
| Lateral Ventricles | 7.167010e-01 | 1.735617e-01 | 4.849337e-01 | 4.716905e-01 | 1.154809e-01 | 2.577221e-01 | 3.51% |
